# The association between cerebrospinal ferritin and soluble triggering receptor expressed on myeloid cells 2 along Alzheimer's continuum

**DOI:** 10.3389/fneur.2022.961842

**Published:** 2022-11-03

**Authors:** Xiaolei Shi, Xiaomei Zhong, Huarong Zhou, Nan Zhou, Yachun Hu, Yuping Ning, Alzheimer's Disease Neuroimaging Initiative

**Affiliations:** ^1^Center for Geriatric Neuroscience, The Affiliated Brain Hospital of Guangzhou Medical University, Guangzhou, China; ^2^Guangdong Engineering Technology Research Center for Translational Medicine of Mental Disorders, Guangzhou, China; ^3^The Affiliated Brain Hospital of Guangzhou Medical University, Guangzhou, China; ^4^Department of Neurology, The Affiliated Brain Hospital of Guangzhou Medical University, Guangzhou, China; ^5^The First School of Clinical Medicine, Southern Medical University, Guangzhou, China

**Keywords:** ferritin, sTrem2, iron, Alzheimer's Disease, neuroinflammation

## Abstract

Brain iron accumulation, which is indicated in the cerebrospinal fluid (CSF) ferritin, is associated with the development of Alzheimer's Disease (AD). Studies have indicated that iron deposition might participate in Alzheimer's pathology through the induction of microglial activation. A soluble triggering receptor expressed on myeloid cells 2 (sTrem2) in CSF is increasingly recognized as a reliable indicator for microglia activity in the brain and participates in the development of neuroinflammation. However, the association between CSF ferritin and sTrem2 under the AD continuum has not been well-established. We enrolled individuals from the Alzheimer's Disease Neuroimaging Initiative (ADNI) database. Participants were classified into healthy controls (HC, *n* = 46) and AD continuum (*n* = 105) in the combined strata of Amyloid/Tau/Neurodegeneration (ATN) mode and Clinical Dementia Rating (CDR) criteria. The associations between CSF ferritin (indicating iron burden) and sTrem2, as well as AD pathology, which is reflected by Aβ42, t-tau, and p-tau in CSF, were explored. CSF ferritin was significantly associated with sTrem2 among all participants (β = 0.517, *P* < 0.001, FDR < 0.001), HC (β = 0.749, *P* = 0.006, FDR = 0.010), and AD continuum (β = 0.488, *P* < 0.001, FDR < 0.001), respectively. However, ferritin predicted the accelerated sTrem2 level in those with high ferritin (β = 0.549, *P* = 0.036, FDR = 0.045). In conclusion, CSF ferritin serves as a potential biomarker of Trem2-indicated microglia function.

## Introduction

Iron homeostasis is pivotal for the operation of the central nervous system (CNS) (1). The metal participates in numerous biological functions of cells, including oxygen transformation, myelin production, as well as the generation and transportation of neurotransmitters (2). Growing evidences have indicated that aging and patients with Alzheimer's Disease (AD), Parkinson's Disease (PD), and amyotrophic lateral sclerosis (ALS) present obvious iron deposition in CNS (3, 4). The excessive iron facilitates the inflammatory responses through the overproduction of hydroxyl radical factors, and induces oxidative stress and apoptosis of the cells in the brain (5).

It has been discovered that iron-positive cells in AD are of a microglial source (6). Microglia, which account for about 5% of the total cells in the brain, are the main immune components (7). Generally, they modify the homeostasis of CNS *via* their intrinsic abilities of secretion, chemotaxis, and clearance. Those activated cells would turn into an inflammatory phenotype in AD, exhibiting the typical morphological changes and producing inflammatory factors (8). The chronic inflammation would exacerbate the deposition of protein aggregates and neuronal loss during progression. A triggering receptor expressed on myeloid cells 2 (Trem2), which is a transmembrane protein on the microglia, is a classical marker for the cells (9). It plays a key role in cell survival, cell proliferation, and immune regulation. It has been discovered that Trem2-related microglia responses may generate neuroprotective effects on AD (10). It contains a long ectodomain that senses the extracellular alterations. Soluble form of Trem2 (sTrem2) is produced through the proteolytic cleavage of the ectodomain by a disintegrin and metalloprotease, and can be identified in cerebrospinal fluid (CSF) (11). It is reported that CSF sTrem2 helps identify the development of AD and is a reliable predictor of AD (12, 13). Therefore, it is important to investigate the association between iron and microglia, as well as their relationships with Alzheimer's pathology.

Moreover, increasing studies have pointed out the importance of understanding AD in a comprehensive manner covering both clinical and pathological changes. An Amyloid/Tau/Neurodegeneration-Clinical Dementia Rating (ATN-CDR) mode was used in this study to evaluate the natural progression process of Alzheimer's dementia, which offers a comprehensive and reasonable way of taking both pathological and clinical presentations into account (14). We used data from the Alzheimer's Disease Neuroimaging Initiative (ADNI) to study the associations of brain iron accumulation (indicated by CSF ferritin) with microglia activities (reflected by CSF sTrem2), based on ATN-CDR mode.

## Methods

### Participants

Data used in the current study were obtained from the ADNI database (adni.loni.usc.edu). Detailed information of ADNI enrollment have been described previously (15). ADNI was launched in 2003, led by Dr. Michael W. Weiner. The primary goal of ADNI has been to combine MRI, PET, biological markers, and clinical and neuropsychological scales to assess the progression of MCI and early AD. The study was approved by the institutional review boards of all participating centers across the USA and Canada. Informed written consent was obtained from all participants. For up-to-date information on ADNI, see www.adni-info.org. The participants included in the current study were available for CSF ferritin, CSF sTrem2, ApoE4 presence, MMSE, and demographic information, including age, gender, and education. Alzheimer's biomarkers, indicated by CSF Aβ42, total-tau (t-tau) and phospho-tau (p-tau), and CDR were acquired to facilitate the clinical-A/T/N classification.

A/T/N scheme is a biomarker-based profile, which aims to facilitate the diagnosis of AD. According to the 2018 NIA-AA “research framework” for the diagnosis of AD (16), the A/T/N scheme included 3 biomarker subgroups: “A” as Aβ aggregation, “T” as tauopathy, and “N” as neurodegeneration. In the current study, we classified each biomarker group as negative (–) or positive (+). Aβ-positive (A+) subjects were those with CSF Aβ42 levels <976.6 pg/ml. Tau-positive (T+) subjects referred to those who had a p-tau > 21.8 pg/ml. Neurodegenerative-positive (N+) individuals were those with t-tau > 245 pg/ml. The CSF biomarker statuses established by these cutoffs were proven to be highly concordant with the PET classification in ADNI (17). The T and N groups were merged to reduce the number of groups to be compared, due to the limited number of participants in each group. Thus, TN-positive (TN+) was defined if either/or neurodegeneration were abnormal.

Then, a combination of the A/T/N biomarker profile and the clinical status (CDR) was used to classify the included participants (14). Clinical status was classified as cognitively unimpaired (CDR = 0), very mild dementia (CDR = 0.5), and mild dementia (CDR = 1). Those who were included in the categories of SNAP (*n* =24), and symptomatic individuals without A+ (*n* = 21) or TN+ (*n* = 43), were excluded from the following analysis. Details of the participants are shown in Table 1.

**Table 1:** The classification of the ADNI participants based on the combination of A/T/N mode and clinical status.

		**Clinical status**		
		**CDR = 0 (cognitively unimpaired)**	**CDR = 0.5 (very mild dementia)**	**CDR = 1 (mild dementia)**
CSF biomarker profile	A**–**/TN**–**	**HC (*****n*** **=** **46)**	*n* = 21	*n* = 0
	A+/TN**–**	**PreAD (*****n*** **=** **12)**	*n* = 33	*n* = 10
	A+/TN+	**PreAD (*****n*** **=** **8)**	**AD (*****n*** **=** **70)**	**AD (*****n*** **=** **15)**
	A**–**/TN+	*n = 8*	*n = 15*	*n = 1*
		*SNAP*		

AD, Alzheimer's Disease; CDR, clinical dementia rating score; HC, healthy control; PreAD, preclinical Alzheimer's Disease; SNAP, suspected non-Alzheimer's pathology.

Bold values means the participants included in the study.

### CSF biomarkers

CSF biomarker profiles were established according to the protocols used in ADNI. CSF sTrem2 was measured based on the MSD platform using the ELISA assay established by the Haass' group (available in MSD-sTREM2CORRECTED.csv in the ADNI database). CSF was measured by the electrochemiluminescence immunoassays Elecsys Aβ42, t-tau, and p-tau on a fully automated Elecsys cobas e 601 instrument (available in UPENNBIOMK9.csv in the ADNI database). Details of the CSF biomarkers assessments can be found online in the ADNI database (https://ida.loni.usc.edu).

### Cognitive assessments

Cognitive performances were assessed using a standardized neuropsychological evaluation with a minimum interval of 6 months. MMSE was used to evaluate the global cognition (18). Tests for the memory domain were used to derive a composite memory score-ADNI-MEM (19), including the Rey Auditory Verbal Learning Test (20), AD Assessment Scale-Cognitive Subscale (21), Word Recall of the MMSE (18) and the Wechsler Logical Memory Scale II (22).

### Statistical analysis

Analyses were performed using SPSS for windows version 24.0 (IBM). Baseline characteristics between HC and AD continuum were compared. All analyses were done on all individuals and separate population using analyses of variance (ANOVA) for continuous data and chi-square tests for categorical measures. CSF Aβ42, t-tau, p-tau, ferritin, and sTrem2 were log-transformed to reduce skewness. We analyzed the associations of CSF ferritin with sTrem2 or AD biomarkers (Aβ42, t-tau, p-tau) using linear regression models, with age, gender, and ApoE4 presence as covariates. Moreover, the models were also established to assess the associations of CSF sTrem2 with fluid biomarkers. Subgroup analyses were done on HC, AD continuum, and ferritin positive/negative (based on median level of ferritin: 6.4 ng/ml) populations. Moreover, results were adjusted for multiple testing by calculating false discovery rate (FDR)-corrected *P*+values with the Benjamini-Hochberg method. Statistical significance was set as *P* < 0.05.

## Results

### Characteristics of participants

The study enrolled 151 participants from the ADNI database. Using an established ATN-CDR criteria, they were classified into 46 HC, 20 PreAD, and 85 AD patients. PreAD and AD participants were merged into an AD continuum group (*n* = 105) to limit the number of comparison groups (Table 1). The baseline characteristics of participants are shown in Table 2. There were significant differences between HC and AD continuum, with respect to ApoE4 presence, CSF Aβ42, t-tau, and p-tau (*P* < 0.05).

**Table 2:** Baseline characteristics of the included participants.

	**HC**	**PreAD**	**AD**	**AD continuum**	***P***
No.	46	20	85	105	
Age	75.896 ± 5.013	75.005 ± 5.794	73.357 ± 7.435	73.671 ± 7.156	0.058
Gender (M) (%)	25 (54.35%)	11 (55.00%)	45 (52.94%)	56 (53.33%)	0.525
Education	16.00 (5.00)	16.00 (6.00)	16.00 (5.00)	16.00 (5.00)	0.912
ApoE4 (%)	5 (10.87%)	11 (55.00%)	67 (78.82%)	78 (74.29%)	<0.001^*^
**CSF biomarkers**					
Aβ42	1,437.078 ± 325.203	642.835 ± 153.133	579.155 ± 145.967	591.285 ± 148.743	<0.001^*^
t-tau	201.491 ± 41.778	266.700 ± 83.642	405.078 ± 103.485	379.797 ± 113.328	<0.001^*^
p-tau	17.818 ± 3.704	26.423 ± 9.723	41.694 ± 12.245	38.904 ± 13.190	<0.001^*^
ferritin	6.100 ± 1.750	6.485 ± 2.762	7.408 ± 2.958	7.232 ± 2.932	0.004^*^
sTrem2	4,494.533 ± 2,178.389	4,391.527 ± 2,098.257	4,864.313 ± 2,842.290	4,774.258 ± 2,713.700	0.538
MMSE	29.00 (1.00)	29.00 (2.00)	25.00 (3.00)	26.00 (4.00)	<0.001^*^
ADNI-MEM	0.949 ± 0.549	0.851 ± 0.508	−0.520 ± 0.618	−0.259 ± 0.805	<0.001^*^

AD, Alzheimer's Disease; ADNI, Alzheimer's Disease Neuroimaging Initiative; CSF, cerebrospinal fluid; EF, executive function; HC, healthy control; ICV, intracranial volume; LAN, language; M, male; MCI, mild cognitive impairment; MEM, memory; MMSE, Mini-Mental State Examination.

* *P* < 0.05.

### Associations of CSF ferritin with sTrem2

**Figure 1:**
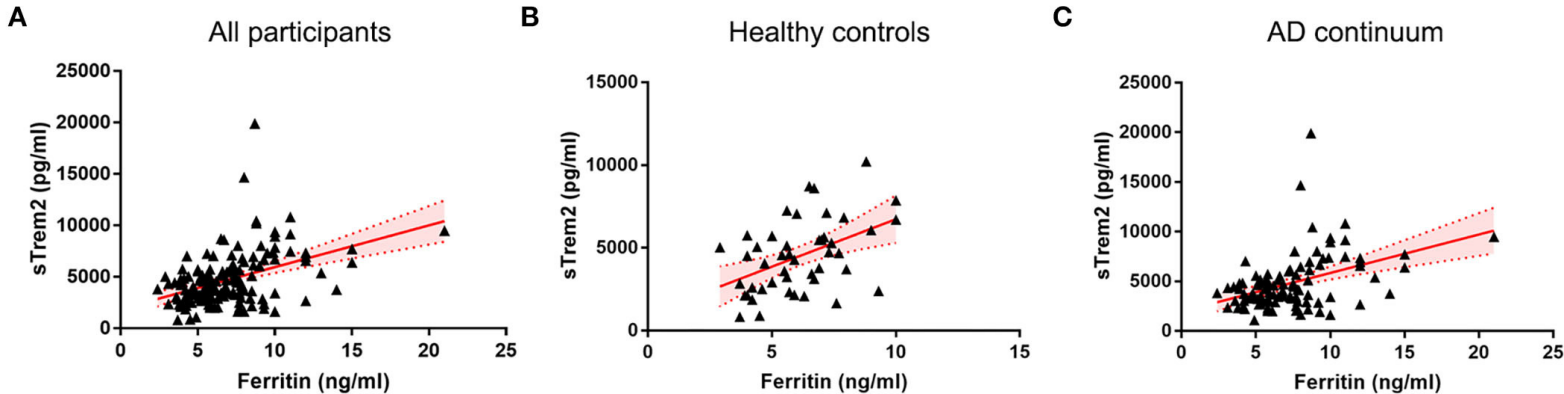
The associations of CSF ferritin with sTrem2 among all participants **(A)**, healthy controls **(B)**, and AD continuum **(C)**. AD, Alzheimer's Disease; CSF, cerebrospinal fluid.

**Figure 2:**
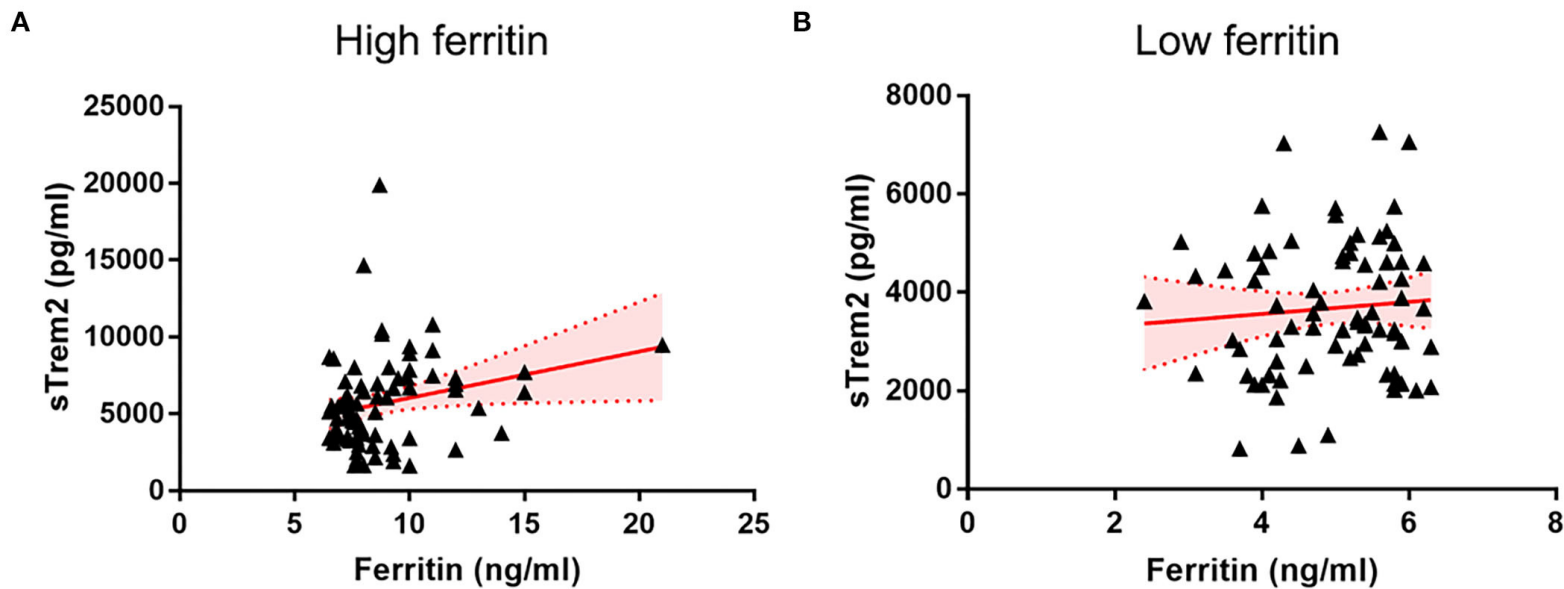
The associations of CSF ferritin with sTrem2 among participants with high **(A)** and low **(B)** CSF ferritin. CSF, cerebrospinal fluid.

In all individuals, higher CSF ferritin, adjusted for age, gender, and ApoE4 presence, was associated with increased sTrem2 (β = 0.517, SE = 0.112, *P* < 0.001, FDR < 0.001) (Supplementary Table 1, Figure 1). The same trends were also found in HC (β = 0.749, SE = 0.258, *P* = 0.006, FDR = 0.010), and patients classified into AD continuum (β = 0.488, SE = 0.124, *P* < 0.001, FDR < 0.001; Figure 1). In median-based analysis (6.4 ng/ml), CSF ferritin was positively correlated with sTrem2 levels in those with high ferritin (β = 0.549, SE = 0.256, *P* = 0.036, FDR = 0.045), but not in participants with low ferritin (β = 0.131, SE = 0.250, *P* = 0.600, FDR = 0.600; Figure 2).

### Associations of CSF ferritin with AD biomarkers

As for AD pathology indicators, no associations were found between CSF ferritin and Aβ42 (β = 0.052, SE = 0.100, *P* = 0.605, FDR = 0.649), t-tau (β = 0.173, SE = 0.089, *P* = 0.054, FDR = 0.138) and p-tau (β = 0.167, SE = 0.102, *P* = 0.104, FDR = 0.260) in any participants (Supplementary Table 1).

We also investigated the predictive values of CSF sTrem2 with AD pathology (Supplementary Table 2). It indicated that sTrem2 is associated with t-tau (β = 0.233, SE = 0.058, *P* < 0.001, FDR < 0.001) and p-tau (β = 0.248, SE = 0.067, *P* < 0.001, FDR = 0.001) levels in all individuals. The significant results were also found in the AD continuum (t-tau: *P* < 0.001, FDR < 0.001; p-tau: *P* < 0.001, FDR < 0.001), but not in healthy participants (*P* > 0.05). Moreover, the status of ferritin burden did not alter the trends of their associations.

## Discussion

The study substantiates the interplay mode between iron and microglia in AD, by demonstrating the positive associations of CSF ferritin with increasing sTrem2 under an evaluation trajectory using an ATN framework and CDR-defined clinical status. Moreover, we investigated the associations of CSF ferritin with neurodegenerative biomarkers, including Aβ42, t-tau, and p-tau across the AD continuum.

sTrem2 in CSF is used to indicate the activity of microglia in the brain (23, 24). The current findings revealed that CSF ferritin was significantly associated with increased sTrem2 among all participants. Furthermore, we found that their links seemed to follow the same pattern in HC and AD continuum. The accordant findings indicated that ferritin in CSF is a potential marker to understanding microglia activity, and supported the upstream role of iron in the induction of neuroinflammation. This is consistent with the previous reports that iron accumulation induces microglia activation, and subsequent inflammatory responses of the cells (6, 25). Kenkhuis et al. (26) found that microglia with increased cellular ferritin light chain, which reflects iron load, show higher Iba-1 expression (a marker of microglia activation), but decreased TMEM119 and P2Y2 receptor expression (both are homeostatic markers), falling into an activated population.

Studies have also indicated that the content of iron increases in the brain of aging populations and patients with AD (4). We tried to explore how the associations of ferritin with microglia would change during the accumulation process, by dividing individuals into high and low ferritin populations with a median cut of CSF ferritin level. Interestingly, we found an unexpected observation that the association between CSF ferritin and sTrem2 existed in individuals with high ferritin, but not in the ones with low ferritin. Taken together, the associations of iron with microglia helps explain the potential disease-modifying effects of iron deposition in brains. High iron storage may trigger the activation of microglia, turning into an iron-dependent activation mode. But the potential mechanism remains unknown.

Of note, an ATN-CDR criterion was used in this study to evaluate the natural progression process of Alzheimer's dementia, which has been proven to offer a comprehensive and reasonable way to take both pathological and clinical presentations into account (14). When we stratified the participants using the classification mode, a significant association of CSF ferritin to t-tau was detected, but not to Aβ42 and p-tau. CSF t-tau is regarded as an indicator for neurodegeneration, from the ATN guideline (16, 27). We may say that CSF ferritin associate with neurodegeneration, but not with Alzheimer's typical pathological conditions. This is not consistent with previous reports that brain iron accumulation accelerates Alzheimer's progression (28). Overload of iron is observed in multiple cortical regions of AD brains. And iron burden was associated with Aβ deposition and tauopathy. Considering the aforementioned link between iron and microglia, we speculated that iron may not accelerate Alzheimer's amyloid and tauopathy in a direct manner.

Although the study added to our understanding of the potential role of CSF ferritin in the context of AD in a clinical setting, there were some limitations in the current project. In this study, brain iron burden was manifested as the ferritin in CSF. Ferritin is the main source of iron in the brain. CSF ferritin should serve as an indirect marker to demonstrate the iron burden of whole brains. Second, the results came from a cross-sectional setting. Further investigation under a longitudinal scope would help the translational application of the findings. Third, more neurodegeneration markers, like neurofilament light chain may be considered for future studies, to ensure the overall analysis of the effects of ferritin on neurodegenerative changes. Moreover, ATN mode is truly a promising evaluation system to understand the progression of AD. Its combination with clinical rating system may need more supporting information.

## Conclusion

In conclusion, we demonstrate that CSF ferritin associates with sTrem2, even in the absence of AD pathology. These findings suggest that CSF ferritin could serve as a surrogate biomarker of Trem2-indicated microglia function.

## Data availability statement

The data analyzed in this study was obtained from the Alzheimer's Disease Neuroimaging Initiative (ADNI), the following licenses/restrictions apply: The application process includes acceptance of the Data Use Agreement and submission of an online application form. The application must include the investigator's institutional affiliation and the proposed uses of the ADNI data. ADNI data may not be used for commercial products or redistributed in any way. Requests to access these datasets should be directed to ADNI, https://adni.loni.usc.edu/data-samples/access-data/.

## Ethics statement

The studies involving human participants were reviewed and approved by Alzheimer's Disease Neuroimaging Initiative (ADNI) and ADNI Data Sharing and Publications Committee. The patients/participants provided their written informed consent to participate in this study.

## Alzheimer's disease neuroimaging initiative

Data used in this article were obtained from the ADNI database (adni.loni.usc.edu), which is easily available for download from the Laboratory of Neuroimaging (LONI) website to the research public. As such, the investigators within the ADNI contributed to the design and implementation of ADNI and/or provided data but did not participate in analysis or writing of this report. A complete listing of ADNI investigators can be found at: http://adni.loni.usc.edu/wp-content/uploads/how-to-apply/ADNI-Acknowledgement-List.pdf.

## Author contributions

YN and XS contributed to the study design and statistical analyses. XS and XZ prepared data. XS, XZ, and HZ drafted the manuscript. NZ and YH participated in data interpretation and manuscript drafting. All authors read and approved the final manuscript.

## Funding

This study was supported by the Science and Technology Project of Guangdong Province (2019B030316001), Guangzhou Municipal Key Discipline in Medicine (2021–2023), National Natural Science Foundation of China (Grant No. 821715333), Foundation of Guangdong Province (2022A1515011623), and Medical Scientific Technology Research Foundation of Guangdong Province (Grant No. A2020446).

## Conflict of interest

The authors declare that the research was conducted in the absence of any commercial or financial relationships that could be construed as a potential conflict of interest.

## Publisher's note

All claims expressed in this article are solely those of the authors and do not necessarily represent those of their affiliated organizations, or those of the publisher, the editors and the reviewers. Any product that may be evaluated in this article, or claim that may be made by its manufacturer, is not guaranteed or endorsed by the publisher.
