# Supplementary material for: The association between cerebrospinal ferritin and soluble triggering receptor expressed on myeloid cells 2 along Alzheimer's continuum
Source: Front Neurol. 2022 Nov 3;13:961842. doi: 10.3389/fneur.2022.961842 (PMC9669339; doi:10.3389/fneur.2022.961842)
Supplement: Supplementary file 1 [file Table_1.docx]

|  |  | **All** | **A-TN-** | **AD continuum** | **ferritin positive** | **ferritin negative** |
| --- | --- | --- | --- | --- | --- | --- |
| **Trem2** | β | 0.517 | 0.749 | 0.488 | 0.549 | 0.131 |
|  | SE | 0.112 | 0.258 | 0.124 | 0.256 | 0.250 |
|  | *P* | <0.001* | 0.006* | <0.001* | 0.036* | 0.600 |
| **Aβ42** | β | 0.052 | 0.105 | 0.036 | -0.090 | 0.164 |
|  | SE | 0.100 | 0.122 | 0.079 | 0.192 | 0.255 |
|  | *P* | 0.605 | 0.398 | 0.649 | 0.647 | 0.521 |
| **tau** | β | 0.173 | 0.134 | 0.179 | 0.042 | 0.357 |
|  | SE | 0.089 | 0.112 | 0.087 | 0.164 | 0.254 |
|  | *P* | 0.054 | 0.239 | 0.042* | 0.800 | 0.164 |
| **p-tau** | β | 0.167 | 0.071 | 0.190 | 0.098 | 0.343 |
|  | SE | 0.102 | 0.111 | 0.098 | 0.190 | 0.289 |
|  | *P* | 0.104 | 0.522 | 0.055 | 0.609 | 0.240 |

**Supplemental Table 1.** The associations of CSF ferritin with Aβ42, tau, p-tau and sTrem2. CSF, cerebrospinal fluid. *P < 0.05.
