# Supplementary material for: The association between cerebrospinal ferritin and soluble triggering receptor expressed on myeloid cells 2 along Alzheimer's continuum
Source: Front Neurol. 2022 Nov 3;13:961842. doi: 10.3389/fneur.2022.961842 (PMC9669339; doi:10.3389/fneur.2022.961842)
Supplement: Supplementary file 2 [file Table_2.docx]

|  |  | **All** | **A-TN-** | **AD continuum** | **ferritin positive** | **ferritin negative** |
| --- | --- | --- | --- | --- | --- | --- |
| **Aβ42** | β | 0.050 | 0.095 | 0.082 | 0.070 | -0.010 |
|  | SE | 0.069 | 0.066 | 0.059 | 0.086 | 0.122 |
|  | *P* | 0.474 | 0.158 | 0.165 | 0.421 | 0.910 |
| **t-tau** | β | 0.233 | 0.116 | 0.255 | 0.168 | 0.322 |
|  | SE | 0.058 | 0.060 | 0.060 | 0.071 | 0.107 |
|  | *P* | <0.001* | 0.061 | <0.001* | 0.021* | 0.004* |
| **p-tau** | β | 0.248 | 0.095 | 0.282 | 0.199 | 0.338 |
|  | SE | 0.067 | 0.059 | 0.067 | 0.082 | 0.123 |
|  | *P* | <0.001* | 0.118 | <0.001* | 0.018* | 0.007* |

**Supplemental Table 2.** The associations of CSF sTrem2 with Aβ42, t-tau, p-tau. CSF, cerebrospinal fluid. *P < 0.05.
